# Supplementary material for: “If I get sick here, I will never see my children again”: The mental health of international migrants during the COVID-19 pandemic in Chile
Source: PLoS One. 2022 Nov 29;17(11):e0277517. doi: 10.1371/journal.pone.0277517 (PMC9707751; doi:10.1371/journal.pone.0277517)
Supplement: S1 File — (PDF) [file pone.0277517.s002.pdf]

GUIÓN DE ENTREVISTA INDIVIDUAL
A personas migrantes internacionales
Versión 01 – 05 de septiembre del 2020
CON TRADUCCIÓN A CREOLE HAITIANO

PROYECTO DE INVESTIGACIÓN

Vulnerabilidades y recursos de comunidades migrantes internacionales en Chile para enfrentar la pandemia SARS-CoV-2: construyendo estrategias diferenciadas desde la interculturalidad

Investigadora principal: Báltica Cabieses, PhD, PROESSA ICIM UDD

1. **Primero una pregunta general sobre la experiencia de migrar**
    - ¿Cómo era su vida antes de migrar? ¿a qué se dedicaba? ¿cómo vivía?
    - ¿Nos puede contar su experiencia de cómo migró a Chile? ¿tuvo alguna experiencia importante para usted? ¿qué le gustó? ¿qué no le gustó de la experiencia? Si pudiera vivirlo de nuevo, ¿qué cambiaría?

2. **Respecto de la experiencia de vivir en su comuna y de trabajar en Chile y efectos de la pandemia**
    - Cuénteme acerca del lugar de residencia donde usted vive, ¿cómo es su vivienda? ¿con quiénes vive?
    - ¿Cómo es el barrio donde usted vive? ¿cómo es su integración a ese barrio? ¿cómo se lleva con sus vecinos?
    - ¿En qué trabaja usted? ¿hace cuánto tiempo? ¿tiene contrato o es informal? ¿tiene medidas de seguridad o protección? ¿cómo se lleva con sus jefaturas y sus pares? ¿Ha cambiado esto con la pandemia?
    - ¿Es su trabajo en Chile similar a su trabajo en su país de origen antes de migrar? ¿De qué manera sus capacidades o estudios previos le han servido para el trabajo que hoy realiza en Chile?

3. **Respecto de experiencias de acceso y uso efectivo del sistema de salud antes y durante la pandemia**
    - ¿Está usted inscrito en el sistema de salud en Chile? ¿cuál sistema previsional de salud tiene? ¿cómo lo eligió? ¿está su familia en Chile (si la tuviera) cubierta también?
    - ¿Ha usado alguna vez el sistema de salud chileno? ¿cuándo, cómo y por qué? ¿cómo evalúa esa experiencia en general? Si pudiera modificar algo del sistema de salud en Chile, ¿qué modificaría?
    - En su opinión, ¿de qué forma la atención de salud en Chile respeta (o no respeta) las diferencias de cultura o cosmovisión de las personas migrantes? ¿qué ejemplos tiene para ilustrar su opinión? ¿y cómo evalúa usted esto?
    - ¿Ha sido alguna vez atendido o apoyado por un facilitador lingüístico o mediador intercultural en su experiencia con el sistema de salud en Chile? ¿cómo fue su experiencia?
    - A propósito de la pandemia por COVID-19, ¿tuvo usted alguna experiencia con esta enfermedad en estos meses? ¿ha podido comprender y seguir las recomendaciones de prevención? ¿usó usted alguna vez el sistema de salud chileno por el COVID-19? ¿cómo fue su experiencia? ¿se siente vulnerable a contagiarse? ¿por qué?

4. **Respecto de recursos y capacidades para enfrentar la pandemia**
    - Para prevenir el contagio por COVID-19 usted y su familia,
        - ¿qué cambios en su vida han hecho? ¿cómo han funcionado dichos cambios? ¿cómo ha logrado implementar esos cambios en la práctica?
        - ¿De qué manera sus redes de apoyo social (pares, amigos, familiares) y de apoyo institucional (municipalidades, juntas de vecinos, equipos de salud, autoridades) han aportado a la prevención de contagio por COVID-19?
    - Para el diagnóstico y tratamiento/recuperación por COVID-19 usted y su familia,
        - ¿Estuvo usted o alguien de su familia enfermo por COVID-19 estos meses? ¿cómo lo enfrentaron? ¿tuvo que residir o conoce a alguien que residió en residencias sanitarias durante estos meses? ¿cómo fue la experiencia?
        - ¿qué cambios en su vida han hecho para el diagnóstico y tratamiento o recuperación? ¿cómo han funcionado dichos cambios? ¿cómo ha logrado implementar esos cambios en la práctica?
        - ¿De qué manera sus redes de apoyo social (pares, amigos, familiares) y de apoyo institucional (municipalidades, juntas de vecinos, equipos de salud, autoridades) han aportado a su diagnóstico y recuperación de COVID-19?
    - Para manejo de efectos negativos por COVID-19 para usted y su familia,
        - ¿Qué efectos o consecuencias ha dejado en usted y su familia el COVID-19? ¿cómo lo está enfrentando? ¿qué necesita para poder enfrentarlo de mejor manera?

5. **Respecto de recomendaciones de mejora para la atención de salud en Chile a migrantes**
    - En su opinión, ¿cómo se podría brindar una mejor atención de salud a migrantes internacionales en Chile?
    - Si pudiera hacer alguna recomendación para que haya una mejor atención en salud, ¿cuál sería?
    - ¿Hay algo más que quisiera compartir con nosotros?

GUIÓN DE ENTREVISTA INDIVIDUAL
A autoridades, líderes y expertos nacionales
Versión 01 – 05 de septiembre del 2020

PROYECTO DE INVESTIGACIÓN

Vulnerabilidades y recursos de comunidades migrantes internacionales en Chile para enfrentar la pandemia SARS-CoV-2: construyendo estrategias diferenciadas desde la interculturalidad

Investigadora principal: Báltica Cabieses, PhD, PROESSA ICIM UDD

1. **Primero una pregunta general sobre la pandemia en Chile**
   - ¿Cómo ha enfrentado nuestro país la pandemia por COVID-19 en estos meses? ¿qué considera que fue acertado o un éxito en este enfrentamiento nacional? ¿qué considera que fue inadecuado o un fracaso en este enfrentamiento nacional? Si pudiera vivirlo de nuevo, ¿qué cambiaría de la forma en la que Chile lo enfrentó?

2. **Respecto de las condiciones de vida y trabajo de migrantes en Chile antes y durante la pandemia**
   - ¿Cómo viven los migrantes en Chile? ¿condiciones materiales de vivienda? ¿hacinamiento? ¿integración barrial y convivencia en nuestro país? ¿protección social y de salud?
   - ¿En qué trabajan principalmente las personas migrantes en Chile? En general ¿tienen contrato o es informal? ¿tienen medidas de seguridad o protección? ¿cómo ha cambiado esto con la pandemia?
   - ¿De qué manera las capacidades o estudios previos de migrantes le han servido a Chile para su desarrollo? ¿ha cambiado esto desde la pandemia? ¿han aportado los migrantes en algo en esta pandemia? ¿en qué? ¿han sido un problema los migrantes en esta pandemia? ¿por qué y cómo?

3. **Respecto de experiencias de acceso y uso efectivo del sistema de salud antes y durante la pandemia**
   - ¿Cómo es el acceso a salud de las personas migrantes en Chile? ¿cómo cree que ellos en general evalúan el sistema de salud en Chile, ¿qué se podría mejorar? ¿qué se debería mantener?
   - En su opinión, ¿de qué forma la atención de salud en Chile respeta (o no respeta) las diferencias de cultura o cosmovisión de las personas migrantes? ¿qué ejemplos tiene para ilustrar su opinión? ¿y cómo evalúa usted esto?
   - ¿Conoce usted el trabajo que realizan facilitadores lingüísticos o mediadores interculturales en el sistema de salud en Chile? ¿qué opina?
   - A propósito de la pandemia por COVID-19, ¿cómo evalúa las acciones y recomendaciones de prevención, diagnóstico y tratamiento hacia migrantes en Chile? ¿son los migrantes un grupo más vulnerable al contagio? ¿por qué?

4. **Respecto de recursos y capacidades para enfrentar la pandemia de parte de migrantes internacionales en Chile**
   - Para <u>prevenir el contagio</u> por COVID-19 usted y su familia,
     - ¿qué cambios en su vida han hecho? ¿cómo han funcionado dichos cambios? ¿cómo ha logrado implementar esos cambios en la práctica?
     - ¿De qué manera sus redes de apoyo social (pares, amigos, familiares) y de apoyo institucional (municipalidades, juntas de vecinos, equipos de salud, autoridades) han aportado a la prevención de contagio por COVID-19?
   - Para <u>el diagnóstico y tratamiento/recuperación</u> por COVID-19 usted y su familia,
     - ¿Estuvo usted o alguien de su familia enfermo por COVID-19 estos meses? ¿cómo lo enfrentaron? ¿tuvo que residir o conoce a alguien que residió en residencias sanitarias durante estos meses? ¿cómo fue la experiencia?
     - ¿qué cambios en su vida han hecho para el diagnóstico y tratamiento o recuperación? ¿cómo han funcionado dichos cambios? ¿cómo ha logrado implementar esos cambios en la práctica?
     - ¿De qué manera sus redes de apoyo social (pares, amigos, familiares) y de apoyo institucional (municipalidades, juntas de vecinos, equipos de salud, autoridades) han aportado a su diagnóstico y recuperación de COVID-19?
   - Para <u>manejo de efectos negativos</u> por COVID-19 para usted y su familia,
     - ¿Qué efectos o consecuencias ha dejado en usted y su familia el COVID-19? ¿cómo lo está enfrentando? ¿qué necesita para poder enfrentarlo de mejor manera?

5. **Respecto de recomendaciones de mejora para la atención de salud en Chile a migrantes**
   - En su opinión, ¿cómo se podría brindar una mejor atención de salud a migrantes internacionales en Chile?
   - Si pudiera hacer alguna recomendación para que haya una mejor atención en salud, ¿cuál sería?
   - ¿Hay algo más que quisiera compartir con nosotros?
