# Supplementary material for: “If I get sick here, I will never see my children again”: The mental health of international migrants during the COVID-19 pandemic in Chile
Source: PLoS One. 2022 Nov 29;17(11):e0277517. doi: 10.1371/journal.pone.0277517 (PMC9707751; doi:10.1371/journal.pone.0277517)
Supplement: Attachment — Submitted filename: Response to reviewers.docx [file pone.0277517.s003.docx]

**Response to reviewers**

**Academic editor**

| Comment | Response |
| --- | --- |
| Please put attention regarding the relevance of the research question that guided the study | The wording of the last sentence of the introduction was adjusted to reflect the main objective of the study presented (lines 143-144) and the research question was added to the methods section (lines 163-166). |
| Please do include a more detailed limitations section | The limitations section was improved especially with regards to the implications of carrying out a secondary analysis (lines 734-742). |
| How were verbatim quotes selected? please provide information and justify the quotes inserted.  I suggest further developing the analysis that is made of the testimonies included and if possible, incorporate some that better represent the topics that are discussed. Many are very short and do not present sufficient evidence. Additionally, I suggest not concluding the paragraphs with a testimony. Please develop the analysis after presenting a testimony | We added information on quote selection in the methods section, we followed these criteria: illustrative (provides an explicit example), succinct, and representative (expresses efficiently and faithfully the pattern described). We reported this in the methods section (lines 228-233). Balancing all three criteria was challenging, especially ensuring that quotes remained succinct. Whenever quotes were shortened by removing irrelevant material to ensure succinctness, an ellipsis “(…)” was used, and authenticity was always prioritised in doing so. In order to ensure that some of them are more explicit we have now lengthened them. We also changed complemented the ones already included with additional ones when necessary.  We also added further analytical elements to in relation to the quotes throughout the results section. |
| Eliminate the two initial tables that take up too much space and also have important wording and style details. Please perform an analysis even if it is descriptive of the sociodemographic information and include it in the development of the text. The tables take away a lot of seriousness and quality to the work. | Thank you, the tables were removed and the paragraphs describing the sociodemographic characteristics of the participants were further developed to include the information previously contained in the tables (lines 258-277). |

**Reviewer 1**

| Comment | Response |
| --- | --- |
| From the description of the migrants’ characteristics, they seem diverse in their sociodemographic characteristics. I suggest the authors add this to the analysis: Did socioeconomic position explained some of the outcomes observed? Could experiences of discrimination be different between migrants in higher or lower socioeconomic positions? What about intersectionality (differences by race, national origin, gender)? | Thank you for raising this shortcoming, we have added further analysis on these points in the results section (lines 373-408) and the discussion (lines 644-650, 655-667). |
| Related to my previous comment: the finding that the pandemic resulted in positive mental health outcomes for some is very interesting, but maybe only the more privileged migrants reported positive outcomes. The quote supporting this theme is from an Argentinian man who was university-educated, had a valid residence permit and seemingly a good job. It may be that his ability to enjoy some aspects of the pandemic are related to his socioeconomic position. Did other participants report positive outcomes? Could the authors comment on this, perhaps by developing the phrase “Emphasizing positive outcomes among findings is key to avoid considering international migrants as a homogenously vulnerable group”? | Thank you for your comment, several other participants reported positive outcomes, all of them among the more highly educated, financially stable participants who entered Chile via regular routes, and who, in some case, had access to positive coping mechanisms. We added a supporting quote and further analysis in the results section (lines 413-426) as well as the discussion section (lines 644-650, 655-667). |
| That some interviewees reported mental health problems as a reason for migration is a very interesting finding. I suggest the authors discuss it a little bit more, as it is seldom addressed in studies of migration and mental health. | Thank you, it is indeed an interesting finding and we have added further analysis in the discussion section (lines 644-650). |
| I suggest the authors discuss the similarities and differences between the negative mental health outcomes related by migrants, and what the non-migrant population was experiencing at the time. Fear of job loss and other social determinants probably were highly prevalent among other persons of lower socioeconomic position as well. | Thank you, this is highly relevant, and we have added further analysis in the results section (lines 470-474, 502-512, 521-524) as well as the discussion (lines 674-690). |
| A minor issue: When exactly were the interviews conducted? This information seems missing from the article, and since the pandemic has been going one for a long time, it would be important to know if the interviews were conducted during lockdown periods, before or after vaccination, etc. | Thank you for pointing this out, we added a sentence explaining that the interviews were carried out during November and December 2020 (lines 170-172). |

**Reviewer 2**

| Comment | Response |
| --- | --- |
| There is no goal/objective or research question | Thank you, the wording of the last sentence of the introduction was adjusted to reflect the main objective of the study presented (lines 143-144) and the research question was added to the methods section (lines 163-166). |
| Adding a discussion about how the secondary analysis methods were used is recommended. This is important because the interview guide does seem to be focused on mental health in any of its sections. Some texts that may be helpful:  Heaton, J. (2004). Secondary Analysis of Qualitative Data. Thousand Oaks, CA: Sage doi: 10.4135/9781446212165.n30.  Ruggiano, N., y Perry, T. E. (2019). Conducting secondary analysis of qualitative data: Should we, can we, and how? Qual Soc Work 18, 81–97. doi: 10.1177/1473325017700701. | Thank you very much for the suggestion, we have added a discussion on secondary analysis in the methods section (lines 156-165, 219-224, 253-256), which we believe enhanced the overall quality of the manuscript. |
| Some questions to address: Did the authors did a recodification? How do they analyze mental health in the secondary study? How does categories or themes in where developed? | Thank you, we have addressed these questions in the methods section (lines 219-224). |
| Authors mentioned that “Figure 1 describes the emerging categories, main categories, generic categories and subcategories from the analysis process as described in the methods section.” This is not clear in the figure. | The wording and figure were modified in order to ensure alignment and clarity (lines 228-23). |
| The number of participants seems to be too high for a case study, for example case studies focus in generating in depth and extensive knowledge, which may not be the case as the collection methods implied 45 min long interviews. Explaining how the authors decided on “saturation” and including the research question may help to understand the study design.  Creswell, W. J., & Creswell, D. J (2018). Research design: Qualitative, quantitative, and mixed methods approach. Sage publications. | This was a collective case study, additionally, the study was exploratory, designed relatively early during the pandemic and carried out when there was still limited information on the situation. In that sense, we respectfully argue that the number of participants and the interviews carried out fit the purpose of the study.  We have included a discussion on saturation in the methods section (lines 212-215). We recognize that saturation was reached for the initial study, however, we included in the limitations section of the discussion a sentence on the limitations of conducting a secondary analysis (lines 734-742). |
| Also, to mention the sample criteria for the case study will be helpful especially due to the diversity of the participants (nationality, migratory status, sex, ages). | Thank you, we have now added that information (lines 176-185). |
| Within this diversity of participants, it would be important to address differences inside the sample (sex, country, migratory status) in the analysis and discussion. | Thank you, we have added further analysis on this point throughout the results section and in the discussion. |
| Many of the reported stressors are not due only to the COVID-19 pandemic and this may have to be discussed, ex. discrimination. | Thank you, we strengthened the idea that many stressors are linked to existing patterns of racism, xenophobia, gender and class discrimination that were exacerbated as a result of the pandemic throughout the results section (on discrimination specifically, see lines 533-539) and in the discussion section, (lines 655-667, 674-690. |
| The article may benefit from discussing with other Latin American literature on the subject. | We added literature from Latin America throughout the discussion section. |
